# Synergistic Effects of Probiotics and Lifestyle Interventions on Intestinal Microbiota Composition and Clinical Outcomes in Obese Adults

**DOI:** 10.3390/metabo15020070

**Published:** 2025-01-23

**Authors:** Glauber Pimentel Florêncio, Analicy Rodrigues Xavier, Ana Catarina de Castro Natal, Lorena Prado Sadoyama, Geórgia das Graças Pena, Ralciane de Paula Menezes, Geraldo Sadoyama Leal, Lislei Jorge Patrizzi, Denise Von Dolinger de Brito Röder

**Affiliations:** 1 School of Medicine, Federal University of Uberlândia, Uberlândia 38405-320, MG, Brazil; glauber.florencio@ufu.br (G.P.F.); analicy.xavier@ufu.br (A.R.X.); ana.natal@ufu.br (A.C.d.C.N.); lohsadoyama@gmail.com (L.P.S.); 2 Institute of Biomedical Sciences, Federal University of Uberlândia, Uberlândia 38405-318, MG, Brazil; ralciane@ufu.br; 3 Institute of Biotechnology, Federal University of Catalão, Catalão 75704-020, GO, Brazil; sadoyama@ufcat.edu.br; 4 Department of Physiotherapy, Federal University of Triângulo Mineiro, Uberaba 38025-350, MG, Brazil; lislei.patrizzi@uftm.edu.br

**Keywords:** intestinal microbiota, probiotics, obesity, lifestyle habits, weight loss

## Abstract

**Background and objective**: Obesity is a growing global epidemic. The composition of the intestinal microbiota can be influenced by several factors. Studies highlight the role of intestinal bacteria in the pathophysiology of obesity. **Objective**: to investigate whether the use of probiotics, together with healthy lifestyle habits, contributes to intestinal microbiota composition and clinical outcomes in individuals with obesity. **Methods**: A prospective study was conducted with 45 adults with obesity. Participants underwent guidance on healthy lifestyle habits, received a probiotic component containing different microbiological strains and were followed for 60 days. Clinical parameters, body composition, biochemical analysis, and intestinal microbiota assessment were evaluated before and after treatment. **Results**: After 60 days, an analysis of the intestinal microbiota showed an increase in microbial diversity (including alfa and beta) and a better balance between the bacterial phyla Firmicutes and Bacteroidetes. Bacterial strains present in the probiotic were also present in the patients’ intestinal microbiota. A reduction in BMI, fasting glucose, insulin, HOMA-IR, LDL cholesterol, and triglycerides was observed, in addition to an increase in HDL cholesterol, improvement in bowel movement frequency, and stool consistency. **Conclusions**: The treatment with probiotics and healthy lifestyle habits contributed to improving the composition of the intestinal microbiota and clinical outcomes individuals with obesity.

## 1. Introduction

Obesity is a growing global epidemic [1], associated with a wide range of chronic diseases, such as type 2 diabetes, high blood pressure, dyslipidemia, hepatic steatosis, obstructive sleep apnea, mood disorders, and musculoskeletal disorders, in addition to certain types of cancer [2]. These comorbidities represent a significant public health challenge, with negative consequences on the quality of life and life expectancy of affected individuals [1].

According to the WHO, in 2016, 39% of the global adult population was overweight [3,4] and 13% were considered obese [4]. Between 1980 and 2023, the prevalence of obesity in the world population tripled [1]. In Brazil, the prevalence of obesity in the adult population increased from 11.8% in 2006 to 22.4% in 2021, according to the Vigitel survey (surveillance of chronic diseases by telephone survey) carried out by the Ministry of Health [5].

Obesity is characterized by an energy imbalance resulting from several factors, such as dysregulated appetite and dysfunction in food reward signaling [1,6]. These changes trigger multiple biological, histological, immunological, and metabolic changes in adipose, liver, muscle, brain, and intestinal tissues [4,7]. It has been observed that the gut microbiome, which consists of trillions of microorganisms, plays an important role in host physiology and is closely linked to obesity [3]. The intestinal microbiome interacts in a complex way with the body, and an imbalance in this microbial community can contribute to the development and progression of obesity [7].

Through advanced omics technologies, such as metagenomics, metatranscriptomics, metaproteomics, and metabolomics, we can analyze in detail this interaction between the microbiome and the host [3,4]. Studies have revealed that healthy individuals have greater bacterial diversity compared to those with high adiposity, insulin resistance, and dyslipidemia, common characteristics in obese patients [7,8]. Furthermore, patients with obesity exhibit a reduced count of bacterial species, indicating a relatively impoverished intestinal microbiota [4,9]. This reduction is associated with a lower proportion of microorganisms from the Bacteroidetes phylum and higher levels of the Firmicutes phylum [4].

However, the composition of the intestinal microbiota can be influenced by several factors [10]. Healthy eating habits [11,12,13,14], encouraging moderate physical activity [14], careful sleep routine [13], and anxiety management [15] were associated with beneficial changes in the intestinal microbiota, contributing to weight reduction [16].

Studies highlight the role of intestinal bacteria in the pathophysiology of obesity, mainly through involvement in low-grade inflammation associated with intestinal dysbiosis [4]. This occurs when the homeostasis of intestinal bacteria is disturbed, leading to changes in the composition, function, and distribution of microorganisms in the intestine, resulting in a state that predisposes the emergence of intestinal pathogenic conditions [12].

Overall, this imbalance increases Firmicutes species such as *Agathobacter rectalis*, *Blautia coccoides*, *Limosilactobacillus reuteri*, *Hathewaya histolytica*, and *Staphylococcus aureus* [3,4]. On the other hand, there are reports of significant reductions in the relative abundance of several members of the phylum Bacteroidetes, such as *Prevotella* and *Alistipes*, in addition to *Faecalibacterium prausnitzii*, *Lactiplantibacillus plantarum*, *Lacticaseibacillus paracasei*, *Lacticaseibacillus rhamnosus*, and phylum Verrucomicrobia (*Akkermansia muciniphila*) [17,18].

In addition to positive lifestyle habit adjustments, there is a growing body of evidence that probiotics improve, maintain, or restore gut microbiota, thus opening the door to innovative maneuvers targeting microbiota architecture and diversity [3]. Research shows that several probiotics, used alone or in symbiotic combinations, can exert anti-obesity effects through species- and strain-specific mechanisms, such as modulation of the intestinal microbiota, greater satiety, and reduced insulin resistance [4,19,20,21].

Probiotics act through three main mechanisms of action in the treatment of obesity: antagonistic effects on the growth of pathogenic microorganisms and competitive adhesion to the intestinal mucosa and epithelium (antimicrobial activity), increased production of the intestinal mucus layer and reduced intestinal permeability (barrier function), and modulation of the gastrointestinal immune system (immunomodulation) [22].

The probiotic bacteria strains *Bifidobacterium longum*, *Lacticaseibacillus casei*, *Levilactobacillus brevis*, *Lacticaseibacillus rhamnosus*, *Lactobacillus delbrueckii*, *Lactobacillus helveticus*, *Lactococcus lactis*, and *Streptococcus thermophilus* may play a role in aiding weight loss in obese individuals through diverse mechanisms of action [3,4,9,12,18,19,20,22]. These strains can modulate the metabolism of fats, optimizing their breakdown, reducing absorption in the intestine, preventing them from being stored in adipose tissue, promoting their use as an energy source [17,23]. Another notable action is the ability to regulate the inflammatory response in the intestine, reducing low-grade inflammation associated with obesity, which can have a positive impact on metabolism and body weight regulation [24]. Furthermore, probiotic strains can affect satiety and appetite signals by modulating the production of hunger-related hormones, such as ghrelin, promoting a greater feeling of fullness. Thus, these mechanisms can help reduce the desire for high-calorie and unhealthy foods [25].

Although there are data indicating that probiotics may play a role in the treatment of obesity, it is important to highlight the need for more solid and comprehensive research to conclusively evaluate their effectiveness, especially their action in addition to healthy lifestyle habits. Therefore, the objective of this study was to explore the integrated effects of healthy lifestyle interventions and the use of probiotics on gut microbiota composition and clinical parameters, with an emphasis on improving metabolic markers and reducing weight in obese individuals.

## 2. Materials and Methods

### 2.1. Study Design

A prospective quasi-experimental study of the before-and-after type was conducted with patients, including both men and women, treated at a primary health care clinic in Uberlândia, in the state of Minas Gerais, Brazil, from October 2022 to April 2023. Initially, 107 patients were invited to participate. Of these, 45 obese patients, with a BMI ≥ 30 kg/m^2^ and aged 18 years or older, met the inclusion criteria and were included in the study. However, 62 patients were excluded based on the following exclusion criteria: use of probiotics in the last 90 days, continuous use of medications in the last 15 days, use of antibiotics in the last 15 days, clinical evidence of intestinal diseases, or the presence of severe comorbidities such as heart disease, nephropathy, chronic liver diseases, immunodeficiencies, chronic neuropathies, or hospitalization in the last two months.

#### Lifestyle Interventions

Forty-five patients were recruited at their first clinic visit (T0). Patients received guidance on the importance of physical activity, sleep hygiene recommendations, and anxiety management. Patients were not encouraged to change their eating patterns; however, guidance was provided to adopt a healthy diet, particularly avoiding industrialized, processed, and ultra-processed products, increasing water intake and fiber consumption, and avoiding soft drinks, following the criteria of the Dietary Guidelines for the Brazilian Population [26].

Recommendations for physical activity included at least 150 to 300 min per week of moderate-intensity physical activity or 75 to 150 min of vigorous-intensity activity, as described in the literature [27,28]. Practical examples, such as brisk walking, recreational cycling, and active household chores, were shared.

Recommendations for improving sleep quality: cognitive therapy was employed to address incorrect beliefs and attitudes about sleep, combined with additional behavioral interventions such as mindfulness- and light-based therapies for initial or late insomnia [29].

Anxiety management: all participants underwent online cognitive–behavioral therapy, receiving instructions and informational materials. They were contacted weekly by a team member to complete lessons lasting approximately 10 to 15 min, over a total of eight weeks. The lessons included strategies such as motivation and goal setting, increasing physical activity, adopting healthy eating habits with an emphasis on avoiding industrialized, processed, and ultra-processed products, increasing water and fiber intake, and reducing the consumption of soft drinks. Additional lessons addressed overcoming barriers to weight loss, implementing behavioral techniques, managing binge eating and emotional overeating, addressing chaotic and emotional eating patterns, applying structured problem-solving methods, and preventing weight regain or setbacks [30].

In addition to these instructions, patients used a probiotic supplement in the form of a capsule containing a combination of the following bacterial strains: *Bifidobacterium longum*, *Lacticaseibacillus casei*, *Levilactobacillus brevis*, *Lacticaseibacillus rhamnosus*, *Lactobacillus delbrueckii*, *Lactobacillus helveticus*, *Lactococcus lactis*, and *Streptococcus thermophilus* (1 capsule, 10^8^ CFU/capsule/day) for a duration of 60 days. The choice of probiotic strains and dosage was based on previous studies demonstrating their efficacy in modulating the gut microbiota and treating metabolic conditions, such as obesity and insulin resistance [9,31,32]. The probiotics were manufactured by Nutramedic—AGA and AGA comercial EIRELI, Brazil. Patients were monitored by regular phone calls regarding the practice of healthy lifestyle habits, in addition to receiving weekly guidance and encouragement via a multiplatform instant messaging service application for smartphones. All were re-evaluated after 60 days (T1). Patients were monitored by regular phone calls regarding the practice of healthy lifestyle habits, in addition to receiving weekly guidance and encouragement via a multiplatform instant messaging service application for smartphones. All were re-evaluated after 60 days (T1).

Instructions to Patients and Stool Sample Collection. Participants received detailed instructions to ensure adherence to the protocol and to preserve the quality of the collected biological samples. Sterile kits were provided for participants to perform the collection safely and efficiently at home. Participants were instructed to collect samples, preferably in the morning, and to store them in refrigerated containers immediately after collection. For transportation, participants used insulated boxes provided by the study, ensuring sample stability until delivery to the laboratory for processing.

**Figure 1:**
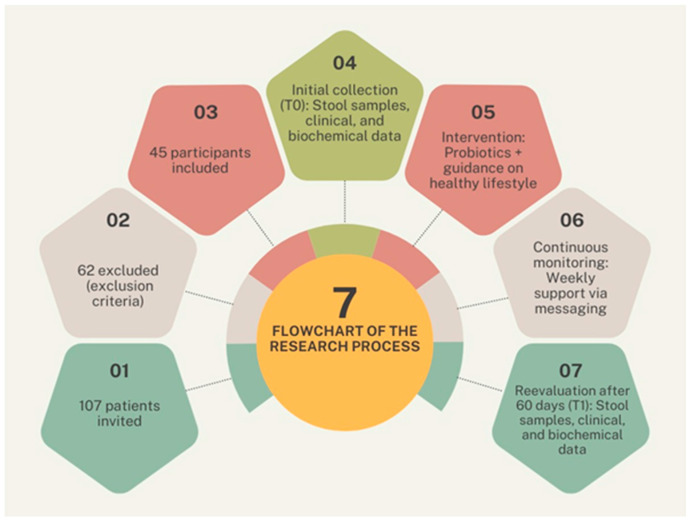
Flowchart of the methodological process detailing the stages of participant selection, intervention, and data collection. It includes the following: (1) initial recruitment of 107 patients; (2) exclusion of 62 participants based on exclusion criteria; (3) inclusion of 45 eligible participants; (4) baseline data collection (T0), including fecal sample and clinical data analysis; (5) implementation of the intervention combining probiotics and lifestyle guidance; (6) continuous monitoring via weekly messages; and (7) re-evaluation after 60 days (T1), with additional data collection and analysis.

Figure 1 illustrates the flowchart of the research process: from recruitment to evaluation.

### 2.2. Anthropometric and Body Composition Variables and Biochemical Parameters

Body mass index (BMI) was calculated using body weight and height measured with bare feet and minimal clothing, according to the World Health Organization’s definition and classification [33]. Body composition parameters (mass and percentage of body fat and lean mass) were acquired using the Inbody 120 bioimpedance scale (South Korea).

A biochemical assessment through a 12 h fasting blood test was performed at T0 and T1, including the following: fasting blood glucose, HbA1C, insulin, HOMA-IR index, HDL cholesterol, LDL cholesterol, and triglycerides. These analyses were carried out in a certified medical laboratory (Sabin, Uberlândia, Brazil).

### 2.3. Stool Shape and Consistency

The form of stools was analyzed using the Bristol Stool Scale [34].

### 2.4. Life Habits

All patients participated in an interview that included a basic question about their dietary pattern using the question “*Considering a usual week, do you consume (food containing fiber/processed foods/soda consumption) frequently*?” (yes/no). Besides that, water consumption was obtained using the question “*Considering a usual week, do you consume how many cups of water/day*?” (1; 2–5; 6–9; ≥10). The Hamilton anxiety rating scale was performed. The following classification was considered: <7 means no anxiety, 7–14 means temporary anxiety, 15–21 means certain anxiety, 21–29 means obvious anxiety, and >29 means severe anxiety [35,36]. Besides that, participants completed the Pittsburgh Sleep Quality Index (PSQI) [37]. Each component is scored on a scale from 0 to 3, with the total score ranging from 0 to 21, where a higher score describes poorer sleep quality. A total PSQI score greater than 5 has been validated as being highly sensitive and specific in distinguishing good from poor sleepers across several populations. Lastly, the International Physical Activity Questionnaire (IPAQ) was considered according to the physical activity score: sedentary: score zero minutes/week; irregularly active: score between 1 and 149 min/week; regularly active: score between 150 and 999 min/week; very active: score > 1000 min/week [38].

### 2.5. Intestinal Microbiota Analysis

#### 2.5.1. Metagenomic Analysis by DNA Sequencing

DNA from stool samples was isolated as described by HEISEN (2016) [39]. For comprehensive metagenomic analysis, shotgun DNA sequencing of the stools was utilized to assess the taxonomy of the intestinal microbiota. The quality and quantity of DNA samples were checked using a Nanodrop Photometer 2000 (Thermo Scientific, Waltham, MA, USA), and the DNA was sequenced on an Illumina HiSeq 2500 Sequencer (Illumina, San Diego, CA, USA). Samples (50 ng as quantified by Qbit (Thermo Fisher Scientific, Waltham, MA, USA) were processed with the Illumina Nextera DNA Sample Preparation Kit (Illumina, San Diego, CA, USA) according to the manufacturer’s protocol. Sequencing was performed with 2 × 100 nucleotides (paired-end sequencing) in 8 lanes with 300 GB of raw data. On average, sequencing reached 2.1 GB/sample. The samples were sequenced with a sequencing depth of 10.9 million reads per paired-end sequencing file (s = 6.3 million) [40].

#### 2.5.2. Bioinformatics Analysis of Sequencing Data

Raw sequences obtained from metagenomic samples of patients underwent a quality check using FastQC software version 0.12.0 (https://www.bioinformatics.babraham.ac.uk/projects/fastqc/, accessed on 1 June 2023) [41]. The quality check comprised base sequence quality, sequence quality scores, base sequence content, sequence GC content, N base content, sequence length distribution, sequence duplication levels, kmer content, and overrepresented sequences. All samples presented satisfactory values for each parameter tested. Then, the sequences were processed using PRINSEQ to remove low-quality reads, cut poly-Ns, and A/T tails [42]. Each sample was subjected to a BLASTX analysis using an in-house-developed tool (MALT http://ab.inf.uni-tuebingen.de/software/malt/, accessed on 1 June 2023) against the NCBI-NR database with a maximum allowed e-value of 1.0. BLASTX files were imported into MEGAN5 (http://ab.inf.uni-tuebingen.de/software/megan5/, accessed on 1 June 2023) [43]. MEGAN5 clustered reads into taxonomic and functional categories based on BLASTX hits. The minimum bit score used for analysis was 50 and a minimum support of 50 reads for each taxonomic category was used for the LCA algorithm. Ultimately, the reads were assigned to a taxonomic and functional category. On average, about 50% of the readings in each sample were assigned to some category, 79% of them to the genus level and about 61% to the species level. The samples were normalized with respect to each other. Functional annotation of the reads was based on the KEGG library (Kyoto Encyclopedia for Genes and Genomes, http://www.genome.jp/kegg/, accessed on 1 June 2023).

Quality control parameters: All samples underwent checks for base sequence quality, sequence GC content, sequence length distribution, and overrepresented sequences using FastQC. These parameters are detailed in the revised manuscript (line 183 updated).

PRINSEQ validation: PRINSEQ was utilized to preprocess reads by trimming low-quality ends, poly-Ns, and A/T tails. While PRINSEQ is not as commonly used as FastP or Trimmomatic, its functions have been validated in previous studies cited in the updated manuscript.

FastQC post-processing check: Quality metrics were re-evaluated after PRINSEQ trimming to ensure data reliability.

BLAST metrics: The analysis now prioritizes alignment length and percentage identity metrics over bit_score to enhance taxonomic and functional reliability.

#### 2.5.3. Statistical Analysis

A paired sample of n = 45 was calculated with a study power of 81.35% (0.8135), an effect size of 0.38, and α = 0.05. The calculation was carried out using G-power software 3.1.9.7.

Descriptive and inferential statistical analyses were performed. The descriptive analysis was carried out using absolute and relative frequencies for qualitative variables. Quantitative variables were analyzed using their means and standard deviation.

For inferential analysis, the following procedures were employed: for quantitative variables (discrete and continuous), normality tests were initially conducted using the KS and SW tests. Subsequently, a paired Student’s *t*-test was performed. For qualitative variables, McNemar’s test was used for nominal variables, and the Wilcoxon test was applied for ordinal variables. The strength of association between qualitative variables was assessed by calculating the odds ratio, accompanied by the respective 95% confidence interval (CI 95%). A significance value of *p* < 0.05 was used to determine statistically significant differences. The analyses were carried out using IBM SPSS software, version 25.0.

## 3. Results

**Figure 2:**
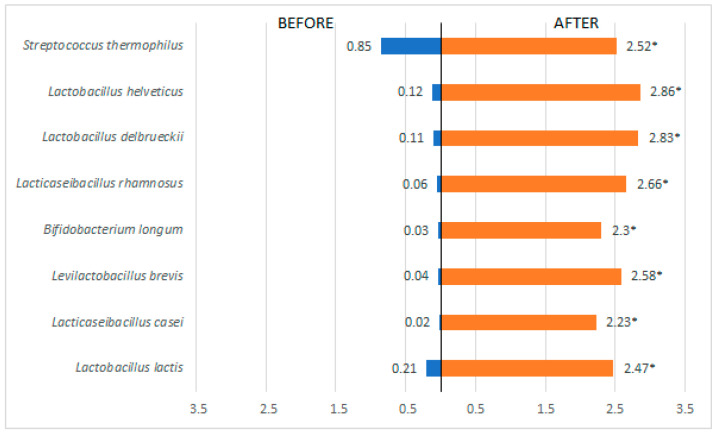
Analysis of the presence of bacterial species of probiotic before and after treatment of the patients included in the study. * *p* < 0.05 = significant difference by the Wilcoxon test.

Of the 45 patients included in the study, 30 (66.7%) were female, with age ranging from 26 to 52 years. All bacterial strains contained in the probiotic used in the treatment were detected in the intestinal microbiota of all patients included in the study (Figure 2).

Table 1 presents the characteristics of the study population in relation to lifestyle habits at time points T0 and T1. An increase in patients without anxiety was observed after treatment, as well as a significant reduction in patients with severe anxiety (from 53.6% to 4.4%), showing 15% more likely to feel without or with temporary anxiety. The percentage of participants classified as highly active increased from 17.8% to 75.6%, while there was a significant reduction in the percentage of participants classified as sedentary, decreasing from 44.4% to 6.7%. Additionally, participants reported a significant improvement in sleep quality, with 24% more likely to achieve a good sleep quality. Regarding the consumption of fiber-rich foods, there was no significant change (*p* = 0.15), with over 85% of patients consuming them both before and after treatment, as the majority already had this dietary habit. As for water intake, there was a significant increase in the daily amount of water consumed by participants after treatment. In terms of soda consumption, there was an increase in the number of patients who stopped consuming this type of beverage, from 33.3% to 73.3%. Regarding the consumption of processed foods, there was a significant reduction in the frequency of these foods’ consumption after treatment, 91.1% to 20.0% (Table 1).

After 60 days, a significant improvement was observed in several physiological parameters (Table 2). An average reduction of 6.04 kg in body weight (*p* < 0.001) and 6.29 kg in fat mass (*p* < 0.001) was observed. The average BMI reduced by 1.1 kg/m^2^ (*p* = 0.003). Fasting glucose and glycated hemoglobin levels showed an average reduction of 8.33 mg/dL and 0.15% (*p* < 0.001). Insulin levels (µUI/mL) reduced by an average of 5.5 (*p* < 0.001). The HOMA-IR index showed an average reduction of 0.99 (*p* = 0.002). Furthermore, an improvement in the lipid profile was observed with a significant mean increase in HDL cholesterol of 8.4 mg/dL (*p* < 0.001) and a mean reduction in LDL cholesterol of 25.69 mg/dL (*p* < 0.001), and the triglyceride levels also decreased by 67.45 mg/dL (*p* < 0.001). Regarding the frequency of evacuation of participants, there was an increase in the frequency of participants who began to evacuate daily from 46.7% to 89.9%. Regarding stool consistency, assessed by the Bristol Scale, there was a 55.3% increase in the percentage of patients with stools classified as type 3 and type 4, compared to the other types from 22.2% to 75.6% with 41% more likely to improve the stool consistency (Table 2).

**Figure 3:**
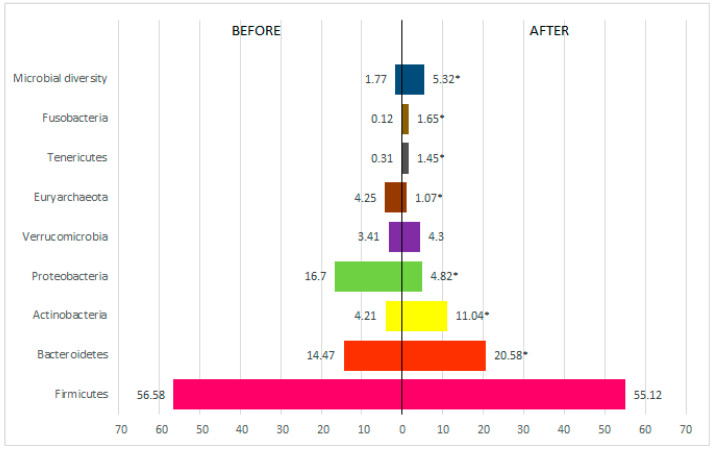
Analysis of phylum-related rates before and after treatment. * *p* < 0.05 = statistically significant difference by the Wilcoxon test.

The results of this study revealed significant changes in the composition of the intestinal microbiota in patients with obesity after 60 days of treatment. Figure 3 shows the rates for the phyla in relation to the period before and after treatment. There was no significant variation in the balance between phyla. A statistically significant increase in the diversity of phyla was observed (*p* < 0.001). The phyla *Proteobacteria* (*p* < 0.001) and *Euryarchaeota* (*p* < 0.001) showed an average reduction. There was no significant difference in the presence of the firmicutes phylum in the pre- and post-treatment periods. The other phyla showed a significant increase in their presence in the intestinal microbiota (*p* < 0.001): *Fusobacteria*, *Tenericutes*, *Euryarchaeota*, *Verrucomicrobia*, *Actinobacteria*, and *Bacteroidetes*.

**Figure 4:**
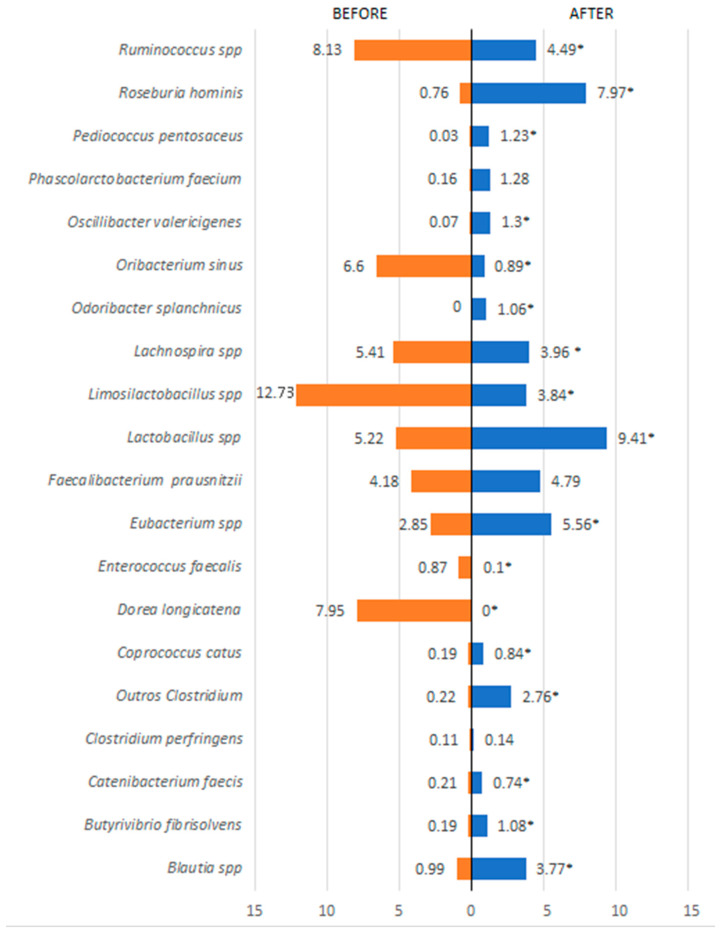
Bacterial species present in the phylum Firmicutes detected before and after treatment. * *p* < 0.05 = a statistically significant difference by the Wilcoxon test.

**Figure 5:**
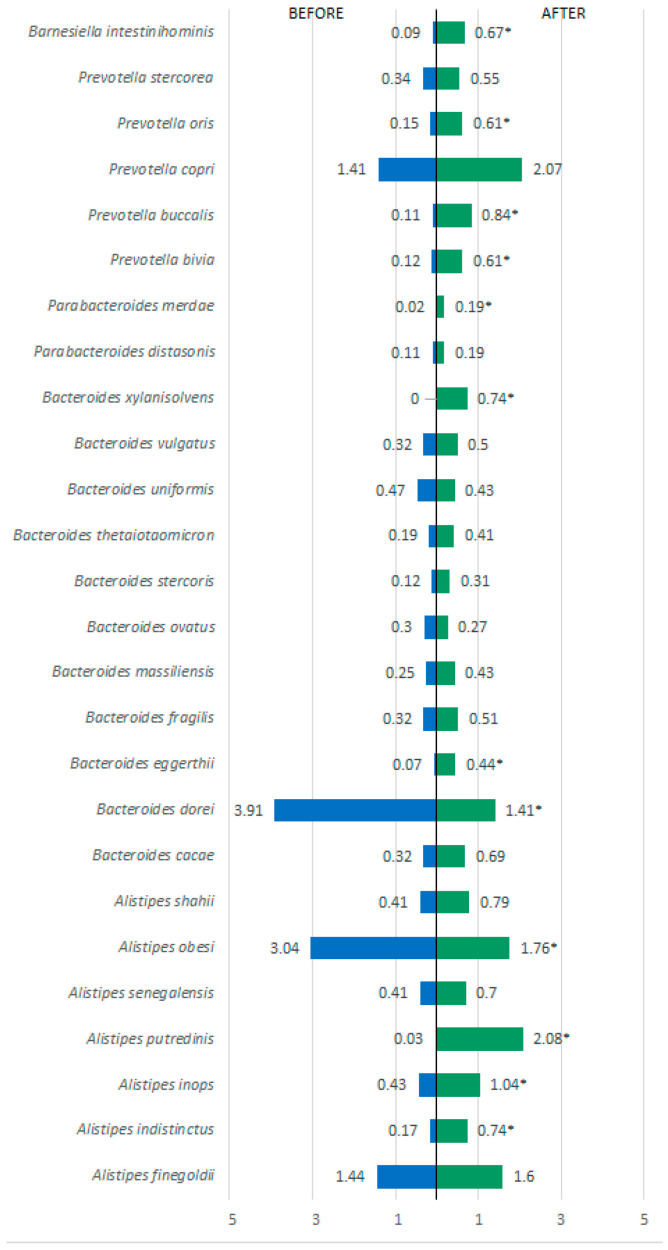
Bacterial species present in the phylum Bacteroidetes detected before and after treatment. * *p* ≤ 0.05 = a statistically significant difference by the Wilcoxon test.

**Figure 6:**
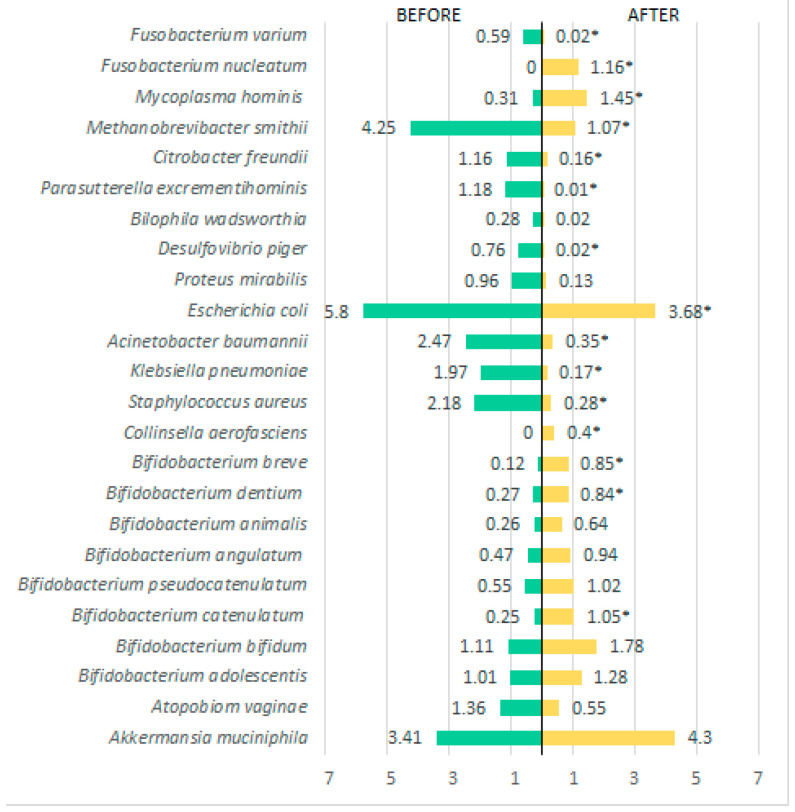
Bacterial species present in the phyla *Verrucomicrobia*, *Actinobacteria*, *Proteobacteria*, *Euryarchaeota*, *Tenericutes*, and *Fusobacteria* detected before and after treatment. * *p* < 0.05 = a statistically significant difference by the Wilcoxon test.

Regarding bacterial species, several showed statistically significant differences (*p* < 0.001) when comparing before and after the 60-day treatment period. In the Firmicutes phylum, there was a reduction in *Ruminococcus* spp., *Oribacterium sinus*, *Lachnospira* spp., *Limosilactobacillus* spp., and *Dorea longicatena*. There was also a significant increase in *Roseburia hominis*, *Lactobacillus* spp., and *Eubacterium* spp. (Figure 4). In the Bacteroidetes phylum (Figure 5), in addition to a substantial increase in diversity, there was a significant increase in various species such as *Barnesiella intestinihominis*, *Alistipes putredini*, and species of *Prevotella*, and a reduction in *Bacteroides dorei* and *Alistipes obesi*. As for other detected phyla, a reduction in their presence in the intestinal microbiota was observed for species such as *Methanobrevibacter smithii* (Euryarchaeota phylum), *Escherichia coli*, *Acinetobacter baumannii*, *Klebsiella pneumoniae*, *Staphyococcus aureus*, *Proteus mirabilis*, *Desulfovibrio piger*, *Bilophila wadsworthia*, *Parasutterella excrementihominis*, and *Citrobacter freundii* (Proteobacteria phylum) (Figure 6).

**Figure 7:**
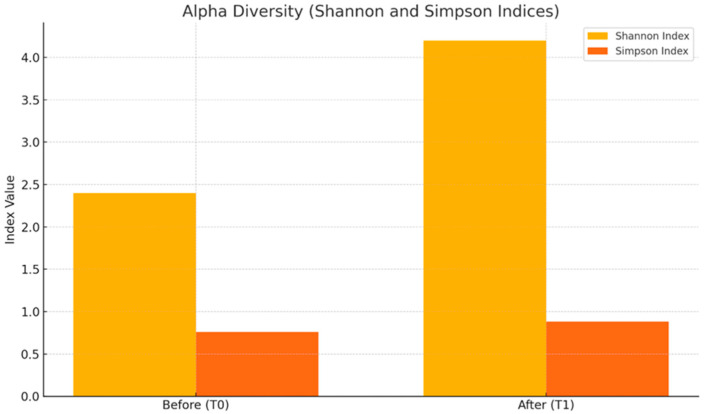
Comparison of alpha diversity metrics: Shannon and Simpson indices pre- and post-intervention. Alpha diversity: displays Shannon and Simpson indices before (T0) and after (T1) the intervention.

Metagenomic analysis revealed significant changes in alpha and beta diversity, as well as important shifts in the taxonomic composition of bacterial phyla. Alpha diversity was assessed using the Shannon and Simpson indices. The Shannon index significantly increased from 2.4 at baseline (T0) to 4.2 after the intervention (T1) (*p* < 0.001), indicating greater species richness and evenness. Similarly, the Simpson index showed a significant improvement, rising from 0.76 to 0.88 (*p* < 0.001), suggesting a reduction in species dominance and a more balanced microbiome (Figure 7).

**Figure 8:**
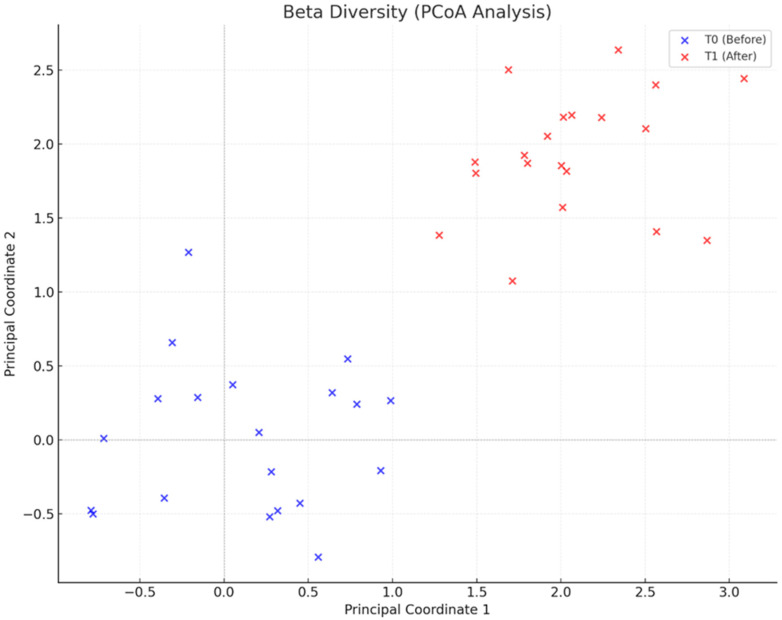
PCoA-based beta diversity analysis: microbial composition shifts between pre- and post-intervention. Beta diversity: depicts the separation of microbial composition through Principal Coordinate Analysis (PCoA) for T0 (before) and T1 (after).

Beta diversity, represented by Principal Coordinate Analysis (PCoA) based on the Bray–Curtis metric, demonstrated a clear separation between the T0 and T1 groups (*p* = 0.01) (Figure 8). This separation reflects significant changes in microbial composition induced by the intervention, indicating favorable remodeling of the gut microbiome. Regarding taxonomic composition, there was a reduction in Firmicutes levels from 56.58% to 55.12% and an increase in Bacteroidetes levels from 14.47% to 20.58% (*p* < 0.05), resulting in a more balanced Firmicutes/Bacteroidetes ratio. These changes were associated with metabolic improvements and a reduction in metabolic dysfunctions. Other phyla, such as Actinobacteria and Verrucomicrobia, showed significant increases, reinforcing microbial diversity and resilience.

At the family level, beneficial species such as *Faecalibacterium prausnitzii* (phylum Firmicutes) and *Akkermansia muciniphila* (phylum Verrucomicrobia) significantly increased after the intervention. Both are recognized for their anti-inflammatory roles and contributions to maintaining intestinal barrier integrity. Conversely, the presence of potentially pathogenic bacteria, such as *Escherichia coli* and *Klebsiella pneumoniae* (phylum Proteobacteria), was significantly reduced. These findings highlight the beneficial effects of combining probiotics with lifestyle interventions, promoting a more balanced and healthier microbiome. Improvements in alpha and beta diversity indices, as well as taxonomic composition changes, corroborate the multifaceted benefits of this integrated approach.

It is noteworthy that, among the 45 patients included in this study, only 5 of them opted exclusively for the use of probiotics, refusing to adhere to the proposal to modify their lifestyle habits, even after continuous stimulation. In these patients, no significant weight loss was observed (with an average loss of only 1 kg over 60 days) and their clinical parameters did not show notable improvements.

All raw data were thoroughly reviewed and validated using SPSS software (version 25.0). Statistical and metagenomic analyses were recalculated to ensure consistency.

## 4. Discussion

This is the first study conducted involving the combined use of a multispecies probiotic and lifestyle changes, with an analysis of the intestinal microbiota, aiming to improve both the intestinal microbiota and various clinical and health parameters, with an emphasis on weight loss. The research involved 45 patients with obesity over a 60-day period and yielded promising results. Previous studies have indicated that the introduction of probiotics can positively influence bacterial composition, resulting in beneficial effects on the health of patients with obesity [15,44,45]. One possible explanation for these effects is related to the increase in short-chain fatty acid (SCFA)-producing bacteria, as well as the reduction in lipopolysaccharide (LPS) producers [7,46,47]. These changes in the gut microbiota have been associated with a reduction in tissue and organ inflammation induced by LPS. Additionally, probiotics may play a role in reducing opportunistic pathogens and their harmful metabolites, such as trimethylamine, LPS, and indole [22]. In the study by Narmaki et al. [48], it was observed that probiotics can reduce fat accumulation, lower inflammation levels, and improve insulin sensitivity [49]. These metabolic benefits have been associated with increased neuropeptides and gastrointestinal peptides, as well as an increase in the abundance of various beneficial bacteria [17,50,51,52,53,54]. However, other studies did not show improvement in weight loss when using probiotics alone [12,55,56]. This underscores that the benefits observed in this study are not attributable to a single isolated factor, but rather to the combined effects of probiotics and healthy lifestyle habits. The synergistic interaction between dietary changes, physical activity, and probiotic supplementation likely creates a more favorable environment for the gut microbiota to exert its metabolic and anti-inflammatory functions, emphasizing the importance of an integrative approach.

There was a significant increase in fatty acids, which play a fundamental role in maintaining intestinal health [54]. These fatty acids have anti-inflammatory effects and can strengthen the integrity of the intestinal barrier, reducing permeability and inflammation [14]. Physical exercise can influence the production of hormones and neurotransmitters that can indirectly affect the intestinal microbiota [55]. In addition to modulating the production of substances such as GABA (gamma-aminobutyric acid) and serotonin, which can influence the composition and function of the microbiota, being of great importance in the homeostasis of the microbiome–gut–brain axis [56]. 

In the present study, the analysis of the degree of anxiety using the Hamilton Anxiety Scale [28] revealed significant improvements and important correlations with the improvement in the intestinal microbiota in obese patients. In this investigation, a significant reduction in anxiety was observed with the positive impact of treatment with probiotics and changes in lifestyle habits, such as practicing meditation, yoga, and deep breathing, on reducing anxiety levels. These results are consistent with previous studies that have shown a correlation between gut health and mental health, highlighting the importance of the gut microbiota in regulating mood and emotional well-being [57,58,59].

The relationship between anxiety and intestinal microbiota has been the subject of increasing interest in the scientific literature. Cai and colleagues demonstrated a bidirectional communication between the brain and the intestine, known as the gut–brain axis, which involves the complex interaction between the central nervous system, the immune system, and the intestinal microbiota [60]. Changes in the gut microbiota have been linked to neuropsychiatric disorders, including anxiety. A balanced gut microbiota is essential for the adequate production of beneficial neurotransmitters, such as GABA and serotonin, which play a crucial role in regulating mood and emotional well-being [15]. The intestinal microbiota is also responsible for the synthesis of vitamins essential for mood, such as vitamin B12 and folate [13]. Therefore, an imbalance in the intestinal microbiota can lead to changes in the production of these neurotransmitters and vitamins, negatively impacting mood and emotional health [23]. The use of probiotics has been suggested as a therapeutic strategy to create a balanced intestinal environment for the production of these essential substances in mental health [61].

Improvement in sleep quality is also a relevant aspect of this study. The reduction in the average Pittsburgh Sleep Quality Index score [29] indicates an overall improvement in participants’ sleep quality. Adequate sleep quality is essential for health and well-being, and sleep disorders are associated with a variety of health problems, including cardiovascular disease, obesity, and cognitive impairment [62,63]. The significant improvement in sleep quality observed in the study may be related to the influence of the intestinal microbiota on the production and regulation of hormones and neurotransmitters involved in sleep, such as melatonin, GABA, and 5-HTP (5-hydroxytryptophan), as has been demonstrated in other research [64,65,66]. Some intestinal microorganisms are capable of synthesizing melatonin from tryptophan, an essential amino acid present in the diet. Therefore, a healthy and diverse gut microbiome can contribute to adequate melatonin production, promoting quality sleep [67,68,69].

When it comes to nutrition, a significant increase in daily water intake was observed after treatment. Adequate hydration is essential for the proper functioning of the body, including gastrointestinal health and the composition of the intestinal microbiota [16,70,71,72]. Additionally, in this research, a significant reduction in the frequency of consumption of industrialized products after treatment was observed. This change is positive, since processed foods are generally rich in saturated fats, sugars, and additives, and their excessive consumption is associated with a greater risk of intestinal dysbiosis, which is associated with obesity, cardiovascular disease, and other chronic conditions [10,73]. Interestingly, in this study, individuals who consumed processed or ultra-processed foods had an increased level of Proteobacteria [74,75,76,77]. Furthermore, regular intake of processed foods is associated with an increase in intestinal permeability, resulting in a condition known as “leaky gut” [23,78,79].

The results obtained in this series demonstrated significant improvements in relation to body mass index (BMI), glycemic control, and lipid profile. The reduction in BMI may be directly related to changes in habits and the improvement in the intestinal microbiota provided by probiotics [9,80]. Furthermore, improvements in glycemic control were observed, with reductions in fasting glucose, glycated hemoglobin, insulin, and HOMA-IR index levels, suggesting an improvement in insulin sensitivity and glycemic control. These results are consistent with previous studies linking metabolic health and gut microbiota composition [81,82,83]. The improvement in the lipid profile was also an important finding. The increase in HDL cholesterol and the reduction in LDL cholesterol and triglycerides indicate a positive effect on the prevention and treatment of participants’ cardiovascular health. These changes are of great relevance, since dyslipidemia is a significant risk factor for cardiovascular diseases [1,84]. There was a significant improvement in participants’ bowel movement frequency after treatment.

The treatment also had a positive effect on participants’ bowel movement frequency and stool consistency. Studies have shown that probiotics can promote the balance of the intestinal microbiota, improving intestinal regularity and stool consistency [3,85,86]. Furthermore, a healthy diet and increased water intake are important factors in maintaining intestinal health. This reinforces the importance of integrative approaches that combine the use of probiotics with lifestyle changes to improve intestinal function and the quality of bowel movements [4,8,87].

Regarding the composition of the intestinal microbiota, the results revealed significant changes with an increase in microbial diversity after treatment. The reduction in the phylum Firmicutes and the increase in the phylum Bacteroidetes stand out, indicating an improvement in the balance between these key bacterial phyla. The reduction in the Firmicutes phylum is particularly relevant, as this group of bacteria is associated with nutrient metabolism and energy extraction from food [11,88]. These changes are consistent with previous studies linking obesity and other metabolic disorders to the imbalance between Firmicutes and Bacteroidetes in the gut microbiota [89]. Although the Firmicutes/Bacteroidetes relationship has often been considered as a possible hallmark of obesity, there is still controversy on this issue due to the relative abundance of the Firmicutes and Bacteroidetes phyla being highly variable among individuals within the same population [87,88,89,90]. This is likely due to many lifestyle factors, including diet, physical activity, food additives and contaminants, antibiotic consumption, and physical activity, among others, which influence the composition of the microbiota in the gastrointestinal tract [2,91,92].

On the other hand, the increase in the Bacteroidetes phylum is associated with a healthier and more diverse microbiota [93]. These bacteria play important roles in the degradation of plant fibers, the production of SCFAs, and the regulation of inflammation in the intestine [92,93,94]. The increase in the Bacteroidetes phylum may contribute to greater SCFA production, which in turn plays a crucial role in maintaining intestinal health by promoting the integrity of the intestinal barrier, regulating the immune response, and providing energy to intestinal cells [18,95,96].

**Figure 9:**
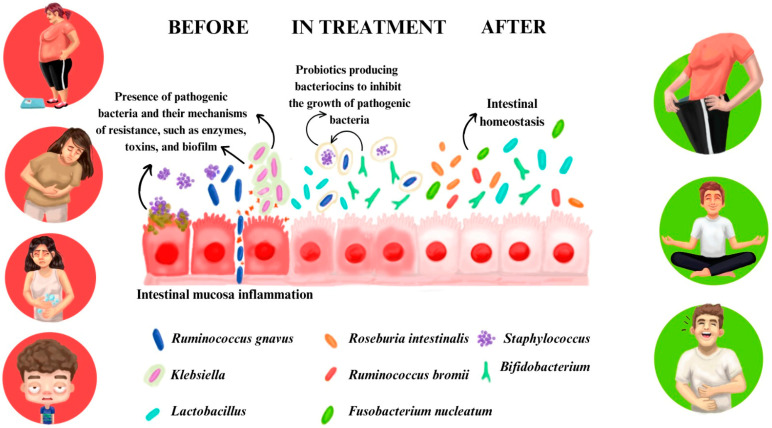
Schematic representation of the evolution of intestinal microbiota during combined probiotics and healthy lifestyle treatment. A representation of a treatment with probiotics.

When analyzing changes in bacterial species, an increase in several beneficial species was observed, such as *Akkermansia muciniphila*, *Prevotella copri*, *Ruminococcus bromii*, *Limosilactobacillus reuteri*, *Bifidobacterium adolescentis*, and *Bifidobacterium bifidum*. These species are associated with health benefits including regulating metabolism, controlling weight, improving the immune system, and reducing inflammation [19,33]. On the other hand, there was a reduction in potentially harmful species, such as *Escherichia coli*, *Bacteroides dorei*, *Lachnospira pectinoschiza*, *Lactobacillus acidophilus*, and *Staphylococcus aureus*. These changes in bacterial species indicate an improvement in the composition of the intestinal microbiota towards a healthier and more balanced profile [21,37,97,98,99,100] (Figure 9 and Supplementary Table).

The first part of the figure (BEFORE) shows the pathogenic bacteria’s resistance mechanisms, such as toxins that cause damage and inflammation of the mucosa. The second part (IN TREATMENT) highlights the benefits of the probiotics, such as bacteriocins, substances that are able to stop the increase in pathogenic bacteria. It is important to notice the reduction in inflammation. The last part (AFTER) shows the mucosa and microbiome after treatment, where the flora is in homeostasis and, as consequence, there is no inflammation in the mucosa.

The exclusive administration of probiotics, without the concomitant implementation of lifestyle changes, results in a considerably less significant response. In our study, patients who followed this approach experienced an average weight loss of 1 kg over 60 days, without significant changes in clinical parameters or in the composition of the intestinal microbiota. These findings highlight the importance of a synergistic approach, where the use of probiotics is combined with the adoption of healthy habits as a more effective strategy to promote meaningful results in weight loss and overall patient well-being.

The robustness of the results presented in our study, compared to the existing literature, can be attributed to the multimodal nature of the intervention. While previous studies focused on the isolated use of probiotics, our work integrated multiple factors known to positively influence the intestinal microbiota, including behavioral changes, anxiety management, and improved sleep quality. This integrated model reflects a more comprehensive strategy capable of optimizing clinical outcomes.

When comparing our findings with the results of the meta-analysis conducted by Borgeraas et al. (2018) [9], we identified important methodological differences. The aforementioned meta-analysis, which evaluated the effect of isolated probiotics without lifestyle interventions, showed modest results, including an average reduction of −0.60 kg in body weight and −0.27 kg/m^2^ in body mass index (BMI). In contrast, our study combined probiotics with dietary guidance, lifestyle changes, and physical activity, producing more expressive and synergistic effects. Additionally, rigorous exclusion criteria were applied to our sample, eliminating potential confounding factors such as recent medication use and the presence of severe comorbidities.

These comparisons reinforce the relevance of integrative approaches that combine probiotics and lifestyle interventions to achieve significant and sustainable clinical benefits. Thus, the results obtained demonstrate that the combination of these strategies is essential to promote substantial improvements in health and patient well-being.

The study has several strengths, including its study design (before-and-after study), a broad panel of measured parameters (anthropometric, biochemical, and lifestyle factors), and microbial analysis of feces demonstrating the influence of probiotic bacteria on the composition of the intestinal microbiota. Additionally, the detailed data collection through in-person meetings, the absence of participant dropouts, and no reported treatment-related side effects are other strong points of the research.

The main limitation of the study is the relatively small number of individuals analyzed. The main reason for this was the use of very strict inclusion and exclusion criteria. However, the criteria applied made it possible to select a homogeneous group of subjects, not affected by diseases or conditions that could have significantly influenced the results of the study. Another limitation was the absence of a control group, which resulted in the risk of overestimating the effectiveness of the treatment and may provide useful insights for future study designs.

## 5. Conclusions

The results of this study indicate that treatment with probiotics and lifestyle modifications for 60 days promoted significant improvements in several clinical and health parameters. These changes are related to improvements in the intestinal microbiota composition, improved eating habits, increased physical activity, reduced anxiety, and better sleep quality. These findings reinforce the importance of an integrated approach to health care, considering not only the intestinal microbiota, but also other aspects of lifestyle. It is recommended that future studies deepen these analyses, including a more detailed assessment of bacterial species and their relationship with different clinical and health parameters, as well as long-term follow-up to assess the sustainability of these improvements.

## Figures and Tables

**Table 1:** Analysis of variables related to lifestyle habits before and after treatment.

Variables	Patients (*n* = 45)		*p* Value *
	Before (T0)	After (T1)	
Anxiety			
Moderate	14 (31.1)	2 (4.4)	<0.001
Severe	24 (53.3)	2 (4.4)	
Temporary	6 (13.3)	16 (35.6)	
No anxiety	1 (2.2)	25 (55.6)	
Physical Activity			
Sedentary	20 (44.4)	3 (6.7)	
Irregularly active	8 (17.8)	0 (0)	
Active	9 (20.0)	8 (17.8)	<0.001
Very active	8 (17.8)	34 (75.6)	
Sleep Quality			
Poor	31 (68.9)	6 (13.3)	
Good	14 (31.1)	39 (86.7)	<0.001
Fiber consumption			
Yes	39 (86.7)	43 (95.6)	
No	6 (13.3)	2 (4.4)	0.125
Water intake (cups/day)			
1	2 (4.4)	6 (13.3)	
2–5	31 (68.9)	5 (11.1)	
6–9	8 (17.8)	11 (24.4)	<0.001
≥10	4 (8.9)	23 (51.1)	
Soda consumption			
Yes	30 (66.7)	12 (26.7)	<0.001
No	15 (33.3)	33 (73.3)	
Processed food consumption			
Yes	41 (91.1)	9 (20.0)	<0.001
No	4 (8.9)	36 (80.0)	

Note: T0 = before treatment; T1 = 60 days after treatment; N (%) = number of patients (percentage); * *p* < 0.05 = a statistically significant difference by the McNemar test (nominal variables).

**Table 2:** Analysis of clinical parameters of patients before and after treatment.

Variables	Patients (*n* = 45) $\bar{x}$ ± SD or *n*(%)		*p* Value *
	Before (T0)	After (T1)	
Weight (kg)	96.67 ± 14.89	90.93 ± 15.01	<0.001
Body fat mass (kg)	39.48 ± 9.58	33.19 ± 8.36	<0.001
Body fat (%)	39.97 ± 8.08	34.30 ± 6.87	<0.001
Lean body mass (kg)	28.59 ± 6.17	29.21 ± 6.42	0.158
BMI (kg/m^2^)	34.63 ± 4.97	33.53 ± 5.35	0.035
Glucose (mmol/L)	95.93 ± 9.86	87.60 ± 6.49	<0.001
HBa1c (%)	5.59 ± 0.45	5.44 ± 0.36	0.010
Insulin (µUI/I)	14.33 ± 7.44	8.83 ± 5.14	<0.001
HOMA-IR	3.36 ± 1.84	2.37 ± 2.65	0.002
HDL (mmol/L)	47.62 ± 12.35	56.02 ± 13.70	<0.001
LDL (mmol/L)	136.36 ± 44.07	110.69 ± 39.76	<0.001
Triglycerides (mmol/L)	171.09 ± 97.66	103.64 ± 46.01	<0.001
BMI classification			
Overweight	0 (0)	13 (28.9)	0.090
Obesity Grade I	34 (75.60)	18 (40)	
Obesity Grade II	6 (13.30)	9 (20)	
Obesity Grade III	5 (11.1)	5 (11.1)	
Bowel movement frequency			
Daily	21 (46.7)	40 (89.9)	<0.001 0.006
Every other day	10 (22.2)	0 (0)	
2 times/week	5 (11.1)	3 (6.7)	
Every 5 days or more	9 (20)	1 (2.2)	
Bristol stool scale			
Others	35 (77.8)	11 (24.4)	<0.001
3 and 4	10 (22.2)	34 (75.6)	

Note: T0 = before treatment; T1 = 60 days after treatment; HBa1c = glycated hemoglobin. BMI = body mass index; HOMA-IR = homeostasis model assessment for insulin resistance; LDL = low-density lipoprotein; HDL= high-density lipoprotein. $\bar{x}\pm \mathrm{SD}$ = (mean ± standard deviation); *n* (%) = number of patients (percentage) * *p* < 0.05 = a statistically significant difference by the McNemar test (nominal variables), the Wilcoxon test (ordinal and continuous variables).

## Data Availability

The data that support the findings of this study are available from the corresponding author (DVDBR), upon reasonable request. The data that support the findings will be available at [https://repositorio.ufu.br/handle/123456789/39223, accessed on 1 May 2023] and [DOI: 10.14393/ufu.di.2023.521] following an embargo from the date of publication to allow for the commercialization of research findings. All data regarding the metagenomic and proteomic analyses conducted by a private company are not available due to patient data protection measures.

## Supplementary Materials

The following supporting information can be downloaded at: https://www.mdpi.com/article/10.3390/metabo15020070/s1, Supplementary Table: Frequency of occurrence of phyla, genera, and species of microorganisms before and after treatment and their role in metabolism (Refs. [101,102,103,104,105,106,107,108,109,110,111,112,113,114,115,116,117,118,119,120,121,122,123,124,125,126,127,128,129,130,131,132,133,134,135,136,137,138,139,140,141,142,143,144,145,146,147,148,149,150,151,152,153,154,155,156,157,158,159,160,161,162,163,164,165,166,167,168,169,170,171,172,173,174,175,176,177,178] are cited in the Supplementary Table).
