# Correction: Florêncio et al. Synergistic Effects of Probiotics and Lifestyle Interventions on Intestinal Microbiota Composition and Clinical Outcomes in Obese Adults. *Metabolites* 2025, *15*, 70

**DOI:** 10.3390/metabo15050293

**Published:** 2025-04-27

**Authors:** Glauber Pimentel Florêncio, Analicy Rodrigues Xavier, Ana Catarina de Castro Natal, Lorena Prado Sadoyama, Geórgia das Graças Pena, Ralciane de Paula Menezes, Geraldo Sadoyama Leal, Lislei Jorge Patrizzi, Denise Von Dolinger de Brito Röder

**Affiliations:** 1 School of Medicine, Federal University of Uberlândia, Uberlândia 38405-320, MG, Brazil; glauber.florencio@ufu.br (G.P.F.); analicy.xavier@ufu.br (A.R.X.); ana.natal@ufu.br (A.C.d.C.N.); lohsadoyama@gmail.com (L.P.S.); 2 Institute of Biomedical Sciences, Federal University of Uberlândia, Uberlândia 38405-318, MG, Brazil; ralciane@ufu.br; 3 Institute of Biotechnology, Federal University of Catalão, Catalão 75704-020, GO, Brazil; sadoyama@ufcat.edu.br; 4 Department of Physiotherapy, Federal University of Triângulo Mineiro, Uberaba 38025-350, MG, Brazil; lislei.patrizzi@uftm.edu.br

Because the corresponding authors found the odds ratio (OR) in the table to be imprecise and unnecessary [1], the authors requested that the OR and text in Tables 1 and 2 be removed. In addition, the authors request to switch the order of Denise Von Dolinger de Brito Röder and Geórgia das Graças Pena. Besides that, we added a reference since we needed to cite the cutoff for the Hamilton Rating Scale for Anxiety (Rabinowitz, et al.).

## Table 1 Legend

In the original publication, there was a mistake in the legend for Table 1. The correct legend appears below. 

Note: T0 = before treatment; T1 = 60 days after treatment; N (%) = number of patients (percentage); * *p* < 0.05 = a statistically significant difference by the McNemar test (nominal variables). 

## Table 2 Legend

In the original publication, there was a mistake in the legend for Table 2.

Note: T0 = before treatment; T1 = 60 days after treatment; HBa1c = glycated hemoglobin. BMI = body mass index; HOMA-IR = homeostasis model assessment for insulin resistance; LDL = low-density lipoprotein; HDL= high-density lipoprotein. $\bar{x}$ ± SD = (mean ± standard deviation); *n* (%) = number of patients (percentage) * *p* < 0.05 = a statistically significant difference by the McNemar test (nominal variables), the Wilcoxon test (ordinal and continuous variables).

## Error in Table 1

In the original publication, there was a mistake in OR analyses as published. We need to withdraw the OR. The corrected table content appears below.

**Table 1:** Analysis of variables related to lifestyle habits before and after treatment.

Variables	Patients (*n* = 45)		*p* Value *
	Before (T0)	After (T1)	
Anxiety			
Moderate	14 (31.1)	2 (4.4)	<0.001
Severe	24 (53.3)	2 (4.4)	
Temporary	6 (13.3)	16 (35.6)	
No anxiety	1 (2.2)	25 (55.6)	
Physical Activity			
Sedentary	20 (44.4)	3 (6.7)	
Irregularly active	8 (17.8)	0 (0)	
Active	9 (20.0)	8 (17.8)	<0.001
Very active	8 (17.8)	34 (75.6)	
Sleep Quality			
Poor	31 (68.9)	6 (13.3)	
Good	14 (31.1)	39 (86.7)	<0.001
Fiber consumption			
Yes	39 (86.7)	43 (95.6)	
No	6 (13.3)	2 (4.4)	0.125
Water intake (cups/day)			
1	2 (4.4)	6 (13.3)	
2–5	31 (68.9)	5 (11.1)	
6–9	8 (17.8)	11 (24.4)	<0.001
≥10	4 (8.9)	23 (51.1)	
Soda consumption			
Yes	30 (66.7)	12 (26.7)	<0.001
No	15 (33.3)	33 (73.3)	
Processed food consumption			
Yes	41 (91.1)	9 (20.0)	<0.001
No	4 (8.9)	36 (80.0)	

## Error in Table 2

In the original publication, there was a mistake in OR analyses as published. We need to withdraw the OR. The corrected table content appears below.

**Table 2:** Analysis of clinical parameters of patients before and after treatment.

Variables	Patients (*n* = 45) $\bar{x}$ ± SD or *n*(%)		*p* Value *
	Before (T0)	After (T1)	
Weight (kg)	96.67 ± 14.89	90.93 ± 15.01	<0.001
Body fat mass (kg)	39.48 ± 9.58	33.19 ± 8.36	<0.001
Body fat (%)	39.97 ± 8.08	34.30 ± 6.87	<0.001
Lean body mass (kg)	28.59 ± 6.17	29.21 ± 6.42	0.158
BMI (kg/m^2^)	34.63 ± 4.97	33.53 ± 5.35	0.035
Glucose (mmol/L)	95.93 ± 9.86	87.60 ± 6.49	<0.001
HBa1c (%)	5.59 ± 0.45	5.44 ± 0.36	0.010
Insulin (µUI/I)	14.33 ± 7.44	8.83 ± 5.14	<0.001
HOMA-IR	3.36 ± 1.84	2.37 ± 2.65	0.002
HDL (mmol/L)	47.62 ± 12.35	56.02 ± 13.70	<0.001
LDL (mmol/L)	136.36 ± 44.07	110.69 ± 39.76	<0.001
Triglycerides (mmol/L)	171.09 ± 97.66	103.64 ± 46.01	<0.001
BMI classification			
Overweight	0 (0)	13 (28.9)	0.090
Obesity Grade I	34 (75.60)	18 (40)	
Obesity Grade II	6 (13.30)	9 (20)	
Obesity Grade III	5 (11.1)	5 (11.1)	
Bowel movement frequency			
Daily	21 (46.7)	40 (89.9)	<0.001 0.006
Every other day	10 (22.2)	0 (0)	
2 times/week	5 (11.1)	3 (6.7)	
Every 5 days or more	9 (20)	1 (2.2)	
Bristol stool scale			
Others	35 (77.8)	11 (24.4)	<0.001
3 and 4	10 (22.2)	34 (75.6)	

## Text Correction 1

There was an error in the original publication. Values with OR.

A correction has been made to Abstract.

**Abstract: Background and objective**: Obesity is a growing global epidemic. The composition of the intestinal microbiota can be influenced by several factors. Studies highlight the role of intestinal bacteria in the pathophysiology of obesity. **Objective**: to investigate whether the use of probiotics, together with healthy lifestyle habits, contributes to intestinal microbiota composition and clinical outcomes in individuals with obesity. **Methods**: A prospective study was conducted with 45 adults with obesity. Participants underwent guidance on healthy lifestyle habits, received a probiotic component containing different microbiological strains and were followed for 60 days. Clinical parameters, body composition, biochemical analysis, and intestinal microbiota assessment were evaluated before and after treatment. **Results**: After 60 days, an analysis of the intestinal microbiota showed an increase in microbial diversity (including alfa and beta) and a better balance between the bacterial phyla Firmicutes and Bacteroidetes. Bacterial strains present in the probiotic were also present in the patients’ intestinal microbiota. A reduction in BMI, fasting glucose, insulin, HOMA-IR, LDL cholesterol, and triglycerides was observed, in addition to an increase in HDL cholesterol, improvement in bowel movement frequency, and stool consistency. **Conclusions**: The treatment with probiotics and healthy lifestyle habits contributed to improving the composition of the intestinal microbiota and clinical outcomes individuals with obesity.

## Text Correction 2

A correction has been made to 2. Materials and Methods, 2.4. Life Habits.

*2.4. Life Habits*

All patients participated in an interview that included a basic question about their dietary pattern using the question “*Considering a usual week, do you consume (food containing fiber/processed foods/soda consumption) frequently*?” (yes/no). Besides that, water consumption was obtained using the question “*Considering a usual week, do you consume how many cups of water/day*?” (1; 2–5; 6–9; ≥10). The Hamilton anxiety rating scale was performed. The following classification was considered: <7 means no anxiety, 7–14 means temporary anxiety, 15–21 means certain anxiety, 21–29 means obvious anxiety, and >29 means severe anxiety [35,36]. Besides that, participants completed the Pittsburgh Sleep Quality Index (PSQI) [37]. Each component is scored on a scale from 0 to 3, with the total score ranging from 0 to 21, where a higher score describes poorer sleep quality. A total PSQI score greater than 5 has been validated as being highly sensitive and specific in distinguishing good from poor sleepers across several populations. Lastly, the International Physical Activity Questionnaire (IPAQ) was considered according to the physical activity score: sedentary: score zero minutes/week; irregularly active: score between 1 and 149 min/week; regularly active: score between 150 and 999 min/week; very active: score > 1000 min/week [38]. 

## Text Correction 3

There was an error in the original publication: Values with OR.

A correction has been made to 3. Results. 

**3. Results**

Of the 45 patients included in the study, 30 (66.7%) were female, with age ranging from 26 to 52 years. All bacterial strains contained in the probiotic used in the treatment were detected in the intestinal microbiota of all patients included in the study (Figure 2).

Table 1 presents the characteristics of the study population in relation to lifestyle habits at time points T0 and T1. An increase in patients without anxiety was observed after treatment, as well as a significant reduction in patients with severe anxiety (from 53.6% to 4.4%), showing 15% more likely to feel without or with temporary anxiety. The percentage of participants classified as highly active increased from 17.8% to 75.6%, while there was a significant reduction in the percentage of participants classified as sedentary, decreasing from 44.4% to 6.7%. Additionally, participants reported a significant improvement in sleep quality, with 24% more likely to achieve a good sleep quality. Regarding the consumption of fiber-rich foods, there was no significant change (*p* = 0.15), with over 85% of patients consuming them both before and after treatment, as the majority already had this dietary habit. As for water intake, there was a significant increase in the daily amount of water consumed by participants after treatment. In terms of soda consumption, there was an increase in the number of patients who stopped consuming this type of beverage, from 33.3% to 73.3%. Regarding the consumption of processed foods, there was a significant reduction in the frequency of these foods’ consumption after treatment, 91.1% to 20.0% (Table 1).

After 60 days, a significant improvement was observed in several physiological parameters (Table 2). An average reduction of 6.04 kg in body weight (*p* < 0.001) and 6.29 kg in fat mass (*p* < 0.001) was observed. The average BMI reduced by 1.1 kg/m^2^ (*p* = 0.003). Fasting glucose and glycated hemoglobin levels showed an average reduction of 8.33 mg/dL and 0.15% (*p* < 0.001). Insulin levels (µUI/mL) reduced by an average of 5.5 (*p* < 0.001). The HOMA-IR index showed an average reduction of 0.99 (*p* = 0.002). Furthermore, an improvement in the lipid profile was observed with a significant mean increase in HDL cholesterol of 8.4 mg/dL (*p* < 0.001) and a mean reduction in LDL cholesterol of 25.69 mg/dL (*p* < 0.001), and the triglyceride levels also decreased by 67.45 mg/dL (*p* < 0.001). Regarding the frequency of evacuation of participants, there was an increase in the frequency of participants who began to evacuate daily from 46.7% to 89.9%. Regarding stool consistency, assessed by the Bristol Scale, there was a 55.3% increase in the percentage of patients with stools classified as type 3 and type 4, compared to the other types from 22.2% to 75.6% with 41% more likely to improve the stool consistency (Table 2).

## References Correction

We need to change the reference to the numbers 35 and 36.

The questions made in the interview did not follow the Elsa questionnaire, but a general question about food patterns. Instead, the reference to the Elsa questionnaire was previously as per [35] (this reference was wrong and should be withdrawn).

The original passage in the original manuscript was as follows: 

*2.4. Life Habits*

All patients participated in an interview that included a dietary pattern survey using the ELSA Brazil questionnaire [35].

The corrected sentence will be as follows (previously presented):

All patients participated in an interview that included a basic question about their dietary pattern using the question “*Considering a usual week, do you consume (food containing fiber/processed foods/soda consumption) frequently*?” (yes/no). Besides that, water consumption was obtained using the question “*Considering a usual week, do you consume how many cups of water/day*?” (1; 2–5; 6–9; ≥10).

The updated references [35,36] will be as follows:

35. Thompson, E. Hamilton Rating Scale for Anxiety (HAM-A). *Occup. Med.* **2015**, *65*, 601. https://doi.org/10.1093/occmed/kqv054.
36. Rabinowitz, J.; Williams, J.B.W.; Hefting, N.; Anderson, A.; Brown, B.; Fu, D.J.; Kadriu, B.; Kott, A.; Mahableshwarkar, A.; Sedway, J.; et al. Consistency checks to improve measurement with the Hamilton Rating Scale for Anxiety (HAM-A). *J. Affect. Disord.* **2023**, *15*, 429–436. https://doi.org/10.1016/j.jad.2023.01.029.

The authors state that the scientific conclusions are unaffected. This correction was approved by the Academic Editor. The original publication has also been updated.
