# Supplementary material for: Synergistic Effects of Probiotics and Lifestyle Interventions on Intestinal Microbiota Composition and Clinical Outcomes in Obese Adults
Source: Metabolites. 2025 Jan 23;15(2):70. doi: 10.3390/metabo15020070 (PMC11857521; doi:10.3390/metabo15020070)
Supplement: Supplementary file 1 [file metabolites-15-00070-s001.zip › metabolites-3363153-supplementary.pdf]

Supplementary table: Frequency of occurrence of phyla, genera, and species of microorganisms before and after treatment and their role in metabolism.

	Before	After	Role in metabolism	Ref.
	N(%)	N(%)		
**Phylum Firmicutes**				
Blautia hydrogenotrophica	10 (22)	36(80)	Involved in regulating the coexistence of anaerobic respiratory pathways.	101
Blautia obeum	3(6)	34(75)	It can inhibit the growth of *C. perfringens* and vancomycin-resistant enterococci, demonstrating its potential as a probiotic with beneficial probiotic effects.	101
Butyrivibrio fibrisolvens	4(8)	28(62)	Lack of information in the literature.	-
Catenibacterium faecis	4(8)	19(42)	Individuals at a heightened risk of cardiovascular disease exhibit decreased levels of *Catenibacterium* species in their microbiota.	102
Clostridium celerecrescens	2(4)	11(24)	Lack of information in the literature.	-
Clostridium coccoides	2(4)	12(26)	There is a positive association between the prevalence of *Clostridium coccoides* and a substantial consumption of monounsaturated fatty acids and polyunsaturated fatty acids.	103
Enterocloster clostridioformis	1(2)	14(31)	Lack of information in the literature.	-
Clostridium fusiformis	1(2)	15(33)	Lack of information in the literature.	-
Clostridium indolis	0	16(35)	Lack of information in the literature.	-
Clostridium perfringens	2(4)	5(11)	*Clostridium perfringens* constitutes a significant factor behind histotoxic and intestinal infections in both humans and other animals.	104
Clostridium phoceensis	2(4)	11(24)	This Family in the gut microbiome is reduced in athletes' metabolism and increased in sedentary people.	105
Coprococcus catus	2(4)	23(51)	Contributes to the production of essential short-chain fatty acids, including butyrate and propionate, which collectively support the well-being of the digestive system and metabolic functions.	106
Dorea longicatena	20(44)	0	Biomarkers of inflammation show a positive connection with *Dorea*	107

			longicatena, indicating its potential role in influencing inflammatory processes.	
Enterococcus faecalis	4(8)	4(8)	This Family might cause infections and is resistant to oxidative stress.	108
Eubacterium contortum	1(2)	10(22)	*Eubacterium contortum* can produce p-cresol, an organic compound associated with health implications.	109
Eubacterium coprostanoligenes	9(20)	0	Found in obese people's microbiome, might decrease cholesterol levels.	110
Eubacterium eligens	2(4)	17(37)	Promising potential of probiotic as a prospective therapeutic focus for addressing atherosclerosis.	111
Dorea formicigenerans	1(2)	24(53)	It has been found to exhibit an inverse correlation with insulin resistance.	107
Eubacterium halii	1(2)	23(51)	It is being assessed in preclinical and clinical trials as potential next-gen probiotic for advancing innovative dietary supplement formulations.	112
Eubacterium ramulus	3(6)	25(55)	Exhibits proficiency in breaking down diverse dietary flavonoids (which provides health benefits).	113
Eubacterium ventriosum	2(4)	22(48)	Demonstrates higher prevalence among individuals with elevated body mass index and serves as a producer of butyrate (molecule that contributes to a healthy gut).	114
Faecalibacterium prausnitzii	13(28)	44(97)	This anti-inflammatory family holds the position of being the most prevalent bacterium in the intestinal microbiota of healthy adults.	115
Limosilactobacillus reuteri	0	36(80)	Describes multiple metabolic pathways that boost the creation of anti-inflammatory cytokines and regulate the gut microbiota through the generation of molecules with antimicrobial properties.	116
Limosilactobacillus fermentum	31(68)	30(66)	Positively influences the host's antioxidant and anti-inflammatory systems, leading to improved glucose regulation in diabetes.	117
Lactobacillus acidophilus	27(60)	32(71)	It can degrade oxalate (a substance that can cause problems) effectively, even when there are other types of carbon sources available that it prefers.	118
Lactobacillus crispatus	5(11)	32(71)	It shows promise as a probiotic option for managing dysbiosis, especially in	119

			women, with potential applications for both prevention and treatment.	
Lactobacillus gasseri	1(2)	32(71)	It can degrade oxalate (substance that can cause problems) effectively, even when there are other types of carbon sources available that it prefers.	118
Lactobacillus ingluviei	35(77)	25(55)	It is related to weight increase.	120
Lactobacillus johnsonii	0	30(66)	It has been extensively researched for its probiotic actions, which involve inhibiting pathogens, attaching to epithelial cells, and modulating the immune system.	121
Lactobacillus rogosae	1(2)	35(77)	It has anti-inflammatory properties and improves insulin sensitivity.	122
Lactobacillus ruminis	5(11)	29(64)	Probiotic resides as a commensal species in the digestive tract and offers potential for application in the functional food field.	123
Lactobacillus sakei	4(8)	32(71)	Exhibits probiotic potential based on in vitro assessment and has the ability to potentially lower inflammation by regulating intestinal metabolism.	124
Lachnospira pectinoschiza	28(62)	17(37)	It's recognized as a type of *Lachnospiraceae* bacteria in the human gut that can make use of pectin, a dietary fiber.	106
Lachnospira multipara	1(2)	28(62)	It is a 2 butyrate producer, a short-chain fatty acid which improves the gut health.	125
Lachnospira straminea	1(2)	24(53)	Lack of information in the literature.	-
Bacillus nealsonii	1(2)	27(60)	Lack of information in the literature.	-
Odoribacter splanchnicus	0	29(64)	Produces short-chain fatty acids. Reduced levels of *Odoribacter* have been linked to microbiota-related diseases, including non-alcoholic fatty liver disease, cystic fibrosis, and inflammatory bowel disease (IBD).	126
Oribacterium sinus	39(86)	15(33)	Lack of information in the literature.	-
Oscillibacter valericigenes	2(4)	32(71)	Is a valerate producer, a type of short-chain fatty acid, generally considered beneficial for gut health.	127
Phascolarctobacterium faecium	2(4)	31(68)	It has the capability to generate beneficial short-chain fatty acids like acetate and propionate, which can promote the well-being of the gut.	128

Pediococcus pentosaceus	1(2)	31(68)	Certain varieties have been documented to alleviate inflammation, encephalopathy, obesity, and fatty liver in animal studies.	129
Roseburia hominis	3(6)	38(84)	Enhances the host's intestinal microbial balance and positively impacts the absorption of nutrients.	130
Roseburia intestinalis	7(15)	38(84)	Demonstrates higher prevalence among individuals with elevated body mass index and serves as a producer of butyrate (molecule that contributes to a healthy gut).	114
Roseburia inulinivorans	3(6)	32(71)	Contributes to the synthesis of butyrate.	131
Roseburia faecis	3(6)	32(71)	Generates short-chain fatty acids, with a particular emphasis on butyrate.	132
Roseburia cecicola	1(2)	34(75)	Also generates short-chain fatty acids, with a particular emphasis on butyrate.	132
Ruminococcus gnavus	34(75)	7(15)	Among adults, *R. gnavus* exhibited the most pronounced responsiveness to disturbances in circadian rhythms.	133
Ruminococcus bromii	3(6)	33(73)	A crucial species responsible for breaking down resistant starch (a substantial energy source) in the human colon.	134
Ruminococcus torques	4(8)	22(48)	Generates an essential enzyme that plays a vital role in the effective production of ursodeoxycholic acid (UDCA), a potent medication used to treat primary biliary cirrhosis and human cholesterol gallstones.	135
**Phylum Bacteroidetes**				
Alistipes finegoldii	17(37)	35(77)	Examination showed a direct link between the prevalence of this group and the acetylation of glycoproteins in overweight women. These glycoproteins have significant roles in biological functions.	136
Alistipes indistinctus	3(6)	23(51)	It exhibits a direct relationship with mast cells and substances that stimulate the immune system.	137
Alistipes inops	7(15)	24(53)	Lack of information in the literature.	-
Alistipes putredinis	1(2)	32(71)	It is a probiotic microorganism that provides advantages to the host organism.	138

Alistipes senegalensis	39(6)	12(26)	Is a common family related to people without obesity.	139
Alistipes obesi	31(68)	37(82)	In lean individuals, it was notably more abundant, and its numbers increased as they pursued their dieting regimen.	140
Alistipes shahii	6(13)	17(37)	Is also a common family related to people without obesity.	139
Bacteroides caccae	4(8)	16(35)	Lack of information in the literature.	-
Bacteroides dorei	39(86)	29(64)	Might decrease the production of lipopolysaccharides by gut microbes and prevent the development of atherosclerosis.	141
Bacteroides eggerthii	3(6)	10(22)	Lack of information in the literature.	-
Bacteroides fragilis	4(8)	10(22)	It serves a significant nutritional function compared to other microorganisms and encourages immune cells to exhibit anti-inflammatory responses.	142
Bacteroides massiliensis	2(4)	11(24)	It has been correlated with distinct probabilities of prostate cancer onset or the degree of prostate cancer progression.	143
Bacteroides ovatus	3(6)	12(26)	The existence of this bacterium appears to elevate immune cell levels, potentially associating it with an increased risk of Type 2 diabetes in obese individuals.	144
Bacteroides stercoris	1(2)	9(20)	Is noticeably more abundant in stool samples from individuals with Diabetic Neuropathy.	145
Bacteroides thetaiotaomicron	3(6)	13(28)	It influences the expression of numerous genes involved in various aspects of the host's physiology, aiding the organism in crucial functions.	146
Bacteroides uniformis	6(13)	14(31)	Lack of information in the literature.	-
Bacteroides vulgatus	5(11)	14(31)	Has the ability to produce GABA.	147
Bacteroides xylanisolvens	0	17(37)	Has the ability to degrade Xylan, a polysaccharide that can serve as a prebiotic.	148
Parabacteroides distasonis	4(8)	7(15)	Aerotolerant anaerobic microbe, exhibiting increasing resistance to antimicrobials, and playing dual roles in human health as both a pathogen and a probiotic.	149

Parabacteroides merdae	1(2)	9(20)	This group is increased in individuals with hypertension.	150
Prevotella bivia	5(11)	15(33)	Lack of information in the literature.	-
Prevotella buccalis	3(6)	21(46)	The changes in the level of IL-1β and TNF-α (immune-related molecules) is associated with this family.	151
Prevotella copri	15(33)	32(71)	It plays a crucial role in the digestive system of many people, making it one of the key components.	152
Prevotella oris	2(4)	15(33)	A strong positive link exists between the occurrence of *P. oris* in the oral microbiota and both a person's age and their level of insulin resistance.	153
Prevotella stercorea	4(8)	13(28)	These bacteria may collaborate with *P. copri* to aid in the digestion of dietary fiber present in our food.	154
Barnesiella intestinihominis	2(4)	19(42)	Is responsible in the gut for amino acid, carbohydrate and fatty acid degradation.	155
**Phylum Actinobacteria**				
Atopobiom vaginae	14(31)	8(17)	It has been demonstrated to have a significant impact on the development and progression of bacterial vaginosis.	156
Bifidobacterium adolescentis	18(40)	27(60)	It is a crucial component of the human gut microbial community, influencing the production of GABA and regulating the communication between the gut and the brain through the gut-brain axis.	157
Bifidobacterium bifidum	21(46)	32(71)	It is more common in infants and is associated with lower occurrence of diarrhea caused by the use of antibiotics.	158
Bifidobacterium catenulatum	7(15)	22(48)	It is a commensal gut bacteria in healthy adults.	159
Bifidobacterium pseudocatenulatum	12(26)	22(48)	Has also the ability to degrade Xylan, a polysaccharide that can serve as a prebiotic.	160
Bifidobacterium angulatum	10(22)	21(46)	This bacterial group's β-galactosidase enzymes break down lactose and foster the growth of beneficial gut bacteria, boosting overall gut health.	161
Bifidobacterium animalis	5(11)	14(31)	Using it as a probiotic supplement is highly promising for supporting obesity treatment.	162
Bifidobacterium dentium	6(13)	18(40)	It has the capacity to process diverse nutrient sources, including many of plant origin, indicating that *B.*	163

			dentium can utilize dietary compounds.	
Bifidobacterium breve	3(6)	17(37)	Is connected to the maintenance of a stable gut microbiome in individuals who are in a healthy condition.	164
Collinsella aerofaciens	0	10(22)	This bacterium's presence is linked to higher secondary bile acid levels, indicating a possible involvement in their production or metabolism in the gut	165
**Phylum Proteobacteria**				
Staphylococcus aureus	21(46)	5(11)	Is a primary reason for biofilm infections on medical equipment, such as prosthetic joints, which impose a substantial healthcare challenge.	166
Klebsiella pneumoniae	13(28)	4(8)	It has the potential to act as a pathogen and a driving factor in the onset of hypertension.	167
Acinetobacter baumannii	14(31)	7(15)	Contributes significantly to the mortality of patients in the intensive care unit (ICU) by causing a variety of infections in this vulnerable ICU population.	168
Escherichia coli	45(100)	44(97)	They are frequently found in the human microbiota, and these isolates can play probiotic, commensal, or pathogenic roles within the host.	169
Proteus mirabilis	9(20)	3(6)	It's recognized for its ability to produce urease and the potential infections it can lead to.	170
Desulfovibrio piger	9(20)	1(2)	The most frequently encountered sulfate-reducing bacteria in the gut within a surveyed group of healthy adults from the United States.	171
Bilophila wadsworthia	5(11)	1(2)	Is able to convert taurine, a common gut substance, into the harmful compound hydrogen sulfide (H2S) by its metabolism. This conversion is linked to inflammatory bowel disease and colorectal cancer.	172
Parasutterella excrementihominis	8(17)	1(2)	The study found that higher levels of *Parasutterella* bacteria in the gut were associated with the activation of a pathway involved in making fatty acids, potentially leading to weight gain. This connection was reinforced when *Parasutterella excrementihominis* levels decreased in participants who followed a low-	173

			carb diet as part of a weight loss program.	
Citrobacter freundii	7(15)	2(4)	*C. freundii,* as an opportunistic pathogen, can lead to a wide range of infections, including those affecting the urinary tract, respiratory tract, wounds, and bloodstream.	174
**Phylum Verrucomicrobia**				
Akkermansia muciniphila	11(24)	45(100)	It is being assessed in preclinical and clinical trials as a potential next-gen probiotic for advancing innovative dietary supplement formulations.	112
**Phylum Euryarchaeota**				
Methanobrevibacter smithii	27(60)	23(51)	Is considered a biomarker that can indicate a healthy colon.	175
**Phylum Tenericutes**				
Mycoplasma hominis	10(22)	37(82)	One of the mycoplasma species that is widely acknowledged for its role in causing the most clinically relevant infections.	176
**Phylum Fusobacteria**				
Fusobacterium nucleatum	0	28(62)	It has been traditionally associated with opportunistic infections. Nevertheless, it is a frequent component of the oral microbiome and can establish a mutually beneficial relationship with its host.	177
Fusobacterium varium	10(22)	1(2)	*F. varium* has been associated with both advantageous and detrimental interactions between bacteria and their host.	178

References:

101- LIU, Xuemei et al. Blautia—a new functional genus with potential probiotic properties?. **Gut microbes**, v. 13, n. 1, p. 1875796, 2021.

102- HOU, Qiangchuan et al. Koumiss consumption modulates gut microbiota, increases plasma high density cholesterol, decreases immunoglobulin G and albumin. **Journal of Functional Foods**, v. 52, p. 469-478, 2019.

103- JAMAR, Giovana et al. Relationship between fatty acids intake and Clostridium coccoides in obese individuals with metabolic syndrome. **Food Research International**, v. 113, p. 86-92, 2018.

104- LI, Jihong; UZAL, Francisco A.; MCCLANE, Bruce A. Clostridium perfringens sialidases: potential contributors to intestinal pathogenesis and therapeutic targets. **Toxins**, v. 8, n. 11, p. 341, 2016.

105- TABONE, Mariangela et al. The effect of acute moderate-intensity exercise on the serum and fecal metabolomes and the gut microbiota of cross-country endurance athletes. **Scientific Reports**, v. 11, n. 1, p. 3558, 2021.

106- VACCA, Mirco et al. The controversial role of human gut lachnospiraceae. **Microorganisms**, v. 8, n. 4, p. 573, 2020.

107- MORTAŞ, Hande; BILICI, Saniye; KARAKAN, Tarkan. The circadian disruption of night work alters gut microbiota consistent with elevated risk for future metabolic and gastrointestinal pathology. **Chronobiology International**, v. 37, n. 7, p. 1067-1081, 2020.

108- KEOGH, Damien et al. Extracellular electron transfer powers Enterococcus faecalis biofilm metabolism. **MBio**, v. 9, n. 2, p. e00626-17, 2018.

109- DU, Hai et al. Exploring the microbial origins of p-cresol and its co-occurrence pattern in the Chinese liquor-making process. **International journal of food microbiology**, v. 260, p. 27-35, 2017.

110- GÉRARD, Philippe. Metabolism of cholesterol and bile acids by the gut microbiota. **Pathogens**, v. 3, n. 1, p. 14-24, 2013.

111- LIU, Sheng et al. Metagenomic analysis of the gut microbiome in atherosclerosis patients identify cross-cohort microbial signatures and potential therapeutic target. **The FASEB Journal**, v. 34, n. 11, p. 14166-14181, 2020.

112- ROMANÍ-PÉREZ, Marina; AGUSTI, Ana; SANZ, Yolanda. Innovation in microbiome-based strategies for promoting metabolic health. **Current Opinion in Clinical Nutrition and Metabolic Care**, v. 20, n. 6, p. 484-491, 2017.

113- BRAUNE, Annett; GÜTSCHOW, Michael; BLAUT, Michael. An NADH-dependent reductase from *Eubacterium ramulus* catalyzes the stereospecific heteroring cleavage of flavanones and flavanonols. **Applied and Environmental Microbiology**, v. 85, n. 19, p. e01233-19, 2019.

114- TIMS, Sebastian et al. Microbiota conservation and BMI signatures in adult monozygotic twins. **The ISME journal**, v. 7, n. 4, p. 707-717, 2013.

115- MIQUEL, Sylvie et al. *Faecalibacterium prausnitzii* and human intestinal health. **Current opinion in microbiology**, v. 16, n. 3, p. 255-261, 2013.

116- ABUQWIDER, Jumana; ALTAMIMI, Mohammad; MAURIELLO, Gianluigi. Limosilactobacillus reuteri in Health and Disease. **Microorganisms**, v. 10, n. 3, p. 522, 2022.

117- LACERDA, Diego Cabral et al. Potential role of *Limosilactobacillus fermentum* as a probiotic with anti-diabetic properties: A review. **World Journal of Diabetes**, v. 13, n. 9, p. 717, 2022.

118- CHAMBERLAIN, Casey A.; HATCH, Marguerite; GARRETT, Timothy J. Metabolomic profiling of oxalate-degrading probiotic *Lactobacillus acidophilus* and Lactobacillus gasseri. **PLos one**, v. 14, n. 9, p. e0222393, 2019.

119- PUEBLA-BARRAGAN, Scarlett et al. Interstrain variability of human vaginal Lactobacillus crispatus for metabolism of biogenic amines and antimicrobial activity against urogenital pathogens. **Molecules**, v. 26, n. 15, p. 4538, 2021.

120- DRISSI, Fatima et al. Comparative genomics analysis of Lactobacillus species associated with weight gain or weight protection. **Nutrition & diabetes**, v. 4, n. 2, p. e109-e109, 2014.

121- PRIDMORE, R. David et al. The genome sequence of the probiotic intestinal bacterium Lactobacillus johnsonii NCC 533. **Proceedings of the National Academy of Sciences**, v. 101, n. 8, p. 2512-2517, 2004.
122- BRERETON, N. J. B.; PITRE, F. E.; GONZALEZ, E. Reanalysis of the Mars500 experiment reveals common gut microbiome alterations in astronauts induced by long-duration confinement. **Computational and Structural Biotechnology Journal**, v. 19, p. 2223-2235, 2021.
123- O'DONNELL, Michelle M. et al. Lactobacillus ruminis strains cluster according to their mammalian gut source. **BMC microbiology**, v. 15, n. 1, p. 1-20, 2015.
124- ZOU, Xuan et al. Probiotic potential of Lactobacillus sakei L-7 in regulating gut microbiota and metabolism. **Microbiological Research**, v. 274, p. 127438, 2023.
125- SHI, Yong et al. Role of gut microbiota in postoperative complications and prognosis of gastrointestinal surgery: a narrative review. **Medicine**, v. 101, n. 29, 2022.
126- HIIPPALA, Kaisa et al. Novel *Odoribacter splanchnicus* strain and its outer membrane vesicles exert immunoregulatory effects in vitro. **Frontiers in microbiology**, v. 11, p. 575455, 2020.
127- PAJARILLO, Edward Alain B. et al. Characterization of the fecal microbial communities of Duroc pigs using 16S rRNA gene pyrosequencing. **Asian-Australasian journal of animal sciences**, v. 28, n. 4, p. 584, 2015.
128- WU, Feifan et al. *Phascolarctobacterium faecium* abundant colonization in human gastrointestinal tract. **Experimental and therapeutic medicine**, v. 14, n. 4, p. 3122-3126, 2017.
129- JIANG, Jie et al. Comparative genomics of *Pediococcus pentosaceus* isolated from different niches reveals genetic diversity in carbohydrate metabolism and immune system. **Frontiers in microbiology**, v. 11, p. 253, 2020.
130- CHENG, Hsin-Lin et al. The next generation beneficial actions of novel probiotics as potential therapeutic targets and prediction tool for metabolic diseases. **Journal of Food and Drug Analysis**, v. 30, n. 1, p. 1, 2022.
131- SCOTT, Karen P. et al. Substrate-driven gene expression in *Roseburia inulinivorans*: importance of inducible enzymes in the utilization of inulin and starch. **Proceedings of the National Academy of Sciences**, v. 108, n. supplement_1, p. 4672-4679, 2011.
132- TAMANAI-SHACOORI, Zohreh et al. *Roseburia* spp.: a marker of health?. **Future microbiology**, v. 12, n. 2, p. 157-170, 2017.
133- CROST, Emmanuelle H. et al. *Ruminococcus gnavus*: friend or foe for human health. **FEMS Microbiology Reviews**, v. 47, n. 2, p. fuad014, 2023.
134- ZE, Xiaolei et al. *Ruminococcus bromii* is a keystone species for the degradation of resistant starch in the human colon. **The ISME journal**, v. 6, n. 8, p. 1535-1543, 2012.
135- ZHENG, Ming-Min et al. Two-step enzymatic synthesis of ursodeoxycholic acid with a new 7β-hydroxysteroid dehydrogenase from Ruminococcus torques. **Process Biochemistry**, v. 50, n. 4, p. 598-604, 2015.

136- LOTANKAR, Mrunalini et al. Distinct Diet-Microbiota-Metabolism Interactions in Overweight and Obese Pregnant Women: a Metagenomics Approach. **Microbiology Spectrum**, v. 10, n. 2, p. e00893-21, 2022.
137- LIU, Jungang et al. Identification of colorectal cancer progression-associated intestinal microbiome and predictive signature construction. **Journal of Translational Medicine**, v. 21, n. 1, p. 1-19, 2023.
138- LIU, Lin et al. Clostridium butyricum potentially improves immunity and nutrition through alteration of the microbiota and metabolism of elderly people with malnutrition in long-term care. **Nutrients**, v. 14, n. 17, p. 3546, 2022.
139- MEIJNIKMAN, A. S. et al. Distinct differences in gut microbial composition and functional potential from lean to morbidly obese subjects. **Journal of Internal Medicine**, v. 288, n. 6, p. 699-710, 2020.
140- JIE, Zhuye et al. The baseline gut microbiota directs dieting-induced weight loss trajectories. **Gastroenterology**, v. 160, n. 6, p. 2029-2042. e16, 2021.
141- YOSHIDA, Naofumi et al. *Bacteroides v*ulgatus and *Bacteroides dorei* reduce gut microbial lipopolysaccharide production and inhibit atherosclerosis. **Circulation**, v. 138, n. 22, p. 2486-2498, 2018.
142- ZAKHARZHEVSKAYA, Natalya B. et al. Outer membrane vesicles secreted by pathogenic and nonpathogenic Bacteroides fragilis represent different metabolic activities. **Scientific reports**, v. 7, n. 1, p. 5008, 2017.
143- SHA, Sybil et al. The human gastrointestinal microbiota and prostate cancer development and treatment. **Investigative and clinical urology**, v. 61, n. Suppl 1, p. S43-S50, 2020.
144- LI, Yue et al. Bacteroides ovatus-mediated CD27− MAIT cell activation is associated with obesity-related T2D progression. **Cellular & Molecular Immunology**, v. 19, n. 7, p. 791-804, 2022.
145- ZHANG, Lili et al. Alterations of the gut microbiota in patients with diabetic nephropathy. **Microbiology Spectrum**, v. 10, n. 4, p. e00324-22, 2022.
146- ZOCCO, Maria Assunta et al. *Bacteroides thetaiotaomicron* in the gut: molecular aspects of their interaction. **Digestive and Liver Disease**, v. 39, n. 8, p. 707-712, 2007.
147- OTARU, Nize et al. GABA production by human intestinal Bacteroides spp.: Prevalence, regulation, and role in acid stress tolerance. **Frontiers in microbiology**, v. 12, p. 656895, 2021.
148- DESPRES, Jordane et al. Xylan degradation by the human gut Bacteroides xylanisolvens XB1AT involves two distinct gene clusters that are linked at the transcriptional level. **BMC genomics**, v. 17, n. 1, p. 1-14, 2016.
149- EZEJI, Jessica C. et al. Parabacteroides distasonis: intriguing aerotolerant gut anaerobe with emerging antimicrobial resistance and pathogenic and probiotic roles in human health. **Gut Microbes**, v. 13, n. 1, p. 1922241, 2021.
150- CUI, Yanlong et al. Roles of intestinal Parabacteroides in human health and diseases. **FEMS microbiology letters**, v. 369, n. 1, p. fnac072, 2022.
151- WANG, Qinghua et al. Multiomics analysis reveals aberrant metabolism and immunity linked gut microbiota with insomnia. **Microbiology Spectrum**, v. 10, n. 5, p. e00998-22, 2022.

152- FRANKE, Thomas; DEPPENMEIER, Uwe. Physiology and central carbon metabolism of the gut bacterium Prevotella copri. **Molecular microbiology**, v. 109, n. 4, p. 528-540, 2018.
153- PRINCE, Yvonne et al. The Relationship between the Oral Microbiota and Metabolic Syndrome. **Biomedicines**, v. 11, n. 1, p. 3, 2022.
154- YEOH, Yun Kit et al. Prevotella species in the human gut is primarily comprised of Prevotella copri, Prevotella stercorea and related lineages. **Scientific Reports**, v. 12, n. 1, p. 9055, 2022.
155- MANCABELLI, Leonardo et al. Meta-analysis of the human gut microbiome from urbanized and pre-agricultural populations. **Environmental microbiology**, v. 19, n. 4, p. 1379-1390, 2017.
156- MENDLING, Werner et al. An update on the role of Atopobium vaginae in bacterial vaginosis: what to consider when choosing a treatment? A mini review. **Archives of gynecology and obstetrics**, v. 300, p. 1-6, 2019.
157- DURANTI, Sabrina et al. Bifidobacterium adolescentis as a key member of the human gut microbiota in the production of GABA. **Scientific reports**, v. 10, n. 1, p. 14112, 2020.
158- O'CALLAGHAN, Amy; VAN SINDEREN, Douwe. *Bifidobacteria* and their role as members of the human gut microbiota. **Frontiers in microbiology**, v. 7, p. 925, 2016.
159- FIRRMAN, Jenni et al. The effect of quercetin on genetic expression of the commensal gut microbes *Bifidobacterium catenulatum*, *Enterococcus caccae* and *Ruminococcus gauvreauii*. **Anaerobe**, v. 42, p. 130-141, 2016.
160- DREY, Elizabeth; KOK, Car Reen; HUTKINS, Robert. Role of Bifidobacterium pseudocatenulatum in Degradation and Consumption of Xylan-Derived Carbohydrates. **Applied and environmental microbiology**, v. 88, n. 20, p. e01299-22, 2022.
161- POKUSAEVA, Karina; FITZGERALD, Gerald F.; VAN SINDEREN, Douwe. Carbohydrate metabolism in Bifidobacteria. **Genes & nutrition**, v. 6, p. 285-306, 2011.
162- MARTORELL, Patricia et al. Probiotic strain Bifidobacterium animalis subsp. lactis CECT 8145 reduces fat content and modulates lipid metabolism and antioxidant response in Caenorhabditis elegans. **Journal of agricultural and food chemistry**, v. 64, n. 17, p. 3462-3472, 2016.
163- ENGEVIK, Melinda A. et al. The metabolic profile of Bifidobacterium dentium reflects its status as a human gut commensal. **BMC microbiology**, v. 21, n. 1, p. 154, 2021.
164- DERRIEN, Muriel et al. Insights into endogenous Bifidobacterium species in the human gut microbiota during adulthood. **Trends in Microbiology**, 2022.
165- BUSTAMANTE, Jessica-Miranda et al. Impact of fecal microbiota transplantation on gut bacterial bile acid metabolism in humans. **Nutrients**, v. 14, n. 24, p. 5200, 2022.
166- BERTRAND, Blake P. et al. Role of Staphylococcus aureus formate metabolism during prosthetic joint infection. **Infection and immunity**, v. 90, n. 11, p. e00428-22, 2022.
167- LI, Jing et al. Causality of opportunistic pathogen Klebsiella pneumoniae to hypertension development. **Hypertension**, v. 79, n. 12, p. 2743-2754, 2022.

168- MORTENSEN, Brittany L.; SKAAR, Eric P. The contribution of nutrient metal acquisition and metabolism to Acinetobacter baumannii survival within the host. **Frontiers in cellular and infection microbiology**, v. 3, p. 95, 2013.
169- GATSIOS, Alexandra; KIM, Chung Sub; CRAWFORD, Jason M. Escherichia coli small molecule metabolism at the host–microorganism interface. **Nature chemical biology**, v. 17, n. 10, p. 1016-1026, 2021.
170- ARMBRUSTER, Chelsie E.; MOBLEY, Harry LT; PEARSON, Melanie M. Pathogenesis of Proteus mirabilis infection. **EcoSal Plus**, v. 8, n. 1, p. 10.1128/ecosalplus. ESP-0009-2017, 2018.
171- REY, Federico E. et al. Metabolic niche of a prominent sulfate-reducing human gut bacterium. **Proceedings of the national academy of sciences**, v. 110, n. 33, p. 13582-13587, 2013.
172- PECK, Spencer C. et al. A glycyl radical enzyme enables hydrogen sulfide production by the human intestinal bacterium Bilophila wadsworthia. **Proceedings of the National Academy of Sciences**, v. 116, n. 8, p. 3171-3176, 2019.
173- HENNEKE, Lea. **Characterization of specific Gut Bacteria and bacterial Metabolites in Human Obesity and Type 2 Diabetes**. 2022. Tese de Doutorado.
174- ANDERSON, Mark T. et al. Citrobacter freundii fitness during bloodstream infection. **Scientific reports**, v. 8, n. 1, p. 11792, 2018.
175- GHAVAMI, Shaghayegh Baradaran et al. Alterations of the human gut Methanobrevibacter smithii as a biomarker for inflammatory bowel diseases. **Microbial pathogenesis**, v. 117, p. 285-289, 2018.
176- POKU, Vivian Osei. Maternal mortality: the role of mycoplasma hominis and its impact on neonatal health. **Health Sciences Review**, p. 100036, 2022.
177- BRENNAN, Caitlin A.; GARRETT, Wendy S. Fusobacterium nucleatum—symbiont, opportunist and oncobacterium. **Nature Reviews Microbiology**, v. 17, n. 3, p. 156-166, 2019.
178- POTRYKUS, Joanna et al. Proteomic investigation of glucose metabolism in the butyrate-producing gut anaerobe Fusobacterium varium. **Proteomics**, v. 7, n. 11, p. 1839-1853, 2007.
